# “Fill States”: PET-derived Markers of the Spatial Extent of Alzheimer Disease Pathology

**DOI:** 10.1148/radiol.241482

**Published:** 2025-03-25

**Authors:** Elena Doering, Merle C. Hoenig, Kathrin Giehl, Verena Dzialas, Grégory Andrassy, Abdelmajid Bader, Andreas Bauer, David Elmenhorst, Johannes Ermert, Silke Frensch, Elena Jäger, Frank Jessen, Philipp Krapf, Tina Kroll, Christoph Lerche, Julia Lothmann, Andreas Matusch, Bernd Neumaier, Oezguer A. Onur, Alfredo Ramirez, Nils Richter, Frederik Sand, Lutz Tellmann, Hendrik Theis, Philip Zeyen, Thilo van Eimeren, Alexander Drzezga, Gérard N. Bischof, for the Alzheimer's Disease Neuroimaging Initiative

**Affiliations:** ^1^Department of Nuclear Medicine, Faculty of Medicine and University Hospital, University of Cologne, Kerpener Str 62, 50937 Cologne, Germany; ^2^German Center for Neurodegenerative Diseases (DZNE), Bonn, Germany; ^3^Institute of Neuroscience and Medicine–Molecular Organization of the Brain (INM-2), Forschungszentrum Jülich, Jülich, Germany; ^4^Faculty of Mathematics and Natural Sciences, University of Cologne, Cologne, Germany; ^5^Department of Psychiatry, Faculty of Medicine and University Hospital, University of Cologne, Cologne, Germany; ^6^Institute of Neuroscience and Medicine–Nuclear Chemistry (INM-5), Forschungszentrum Jülich, Jülich, Germany; ^7^Institute of Neuroscience and Medicine–Imaging-Core-Facility (ICF), Forschungszentrum Jülich, Jülich, Germany; ^8^Institute of Neuroscience and Medicine–Medical Imaging Physics (INM-4), Forschungszentrum Jülich, Jülich, Germany; ^9^Department of Nuclear Chemistry, Faculty of Mathematics and Natural Sciences, University of Cologne, Cologne, Germany; ^10^Faculty of Medicine and University Hospital Cologne, Institute of Radiochemistry and Experimental Molecular Imaging, University of Cologne, Cologne, Germany; ^11^Department of Neurology, Faculty of Medicine and University Hospital, University of Cologne, Cologne, Germany; ^12^Cologne Excellence Cluster for Aging and Aging-Associated Diseases (CECAD), University of Cologne, Cologne, Germany; ^13^Department of Psychiatry and Psychotherapy, Division of Neurogenetics and Molecular Psychiatry, University of Cologne, Medical Faculty, Cologne, Germany; ^14^Department for Neurodegenerative Diseases and Geriatric Psychiatry, University Hospital Bonn, Bonn, Germany; ^15^Department of Psychiatry and Glenn Biggs Institute for Alzheimer’s and Neurodegenerative Diseases, University of Texas Health Science Center at San Antonio, San Antonio, Tex; ^16^Institute of Neuroscience and Medicine–Cognitive Neuroscience (INM-3), Forschungszentrum Jülich, Jülich, Germany

## Abstract

**Background:**

Alzheimer disease (AD) progression can be monitored by tracking intensity changes in PET standardized uptake value (SUV) ratios of amyloid, tau, and neurodegeneration. The spatial extent (“fill state”) of these three hallmark pathologic abnormalities may serve as critical pathophysiologic information, pending further investigation.

**Purpose:**

To examine the clinical utility and increase the accessibility of PET-derived fill states.

**Materials and Methods:**

This secondary analysis of two prospective studies used data from two independent cohorts: the Alzheimer’s Disease Neuroimaging Initiative (ADNI) and the Tau Propagation over Time study (T-POT). Each cohort comprised amyloid-negative cognitively normal individuals (controls) and patients with subjective cognitive decline, mild cognitive impairment, or probable-AD dementia. Fill states of amyloid, tau, and neurodegeneration were computed as the percentages of significantly abnormal voxels relative to controls across PET scans. Fill states and SUV ratios were compared across stages (Kruskal-Wallis *H* test, area under the receiver operating characteristic curve analysis) and tested for association with the severity of cognitive impairment (Spearman correlation, multivariate regression analysis). Additionally, a convolutional neural network (CNN) was developed to estimate fill states from patients’ PET scans without requiring controls.

**Results:**

The ADNI cohort included 324 individuals (mean age, 72 years ± 6.8 [SD]; 173 [53%] female), and the T-POT cohort comprised 99 individuals (mean age, 66 years ± 8.7; 63 [64%] female). Higher fill states were associated with higher stages of cognitive impairment (*P* < .001), and tau and neurodegeneration fill states showed higher diagnostic performance for cognitive impairment compared with SUV ratio (*P* < .05) across cohorts. Similarly, all fill states were negatively correlated with cognitive performance (*P* < .001) and uniquely characterized the degree of cognitive impairment even after adjustment for SUV ratio (*P* < .05). The CNN estimated amyloid and tau accurately, but not neurodegeneration fill states.

**Conclusion:**

Fill states provided reliable markers of AD progression, potentially improving early detection, staging, and monitoring of AD in clinical practice and trials beyond SUV ratio.

Clinical trial registration no. NCT00106899

© The Author(s) 2025. Published by the Radiological Society of North America under a CC BY 4.0 license.

*Supplemental material is available for this article.*

See also the editorial by Yun and Kim in this issue.

## Graphical abstract

**Figure:**
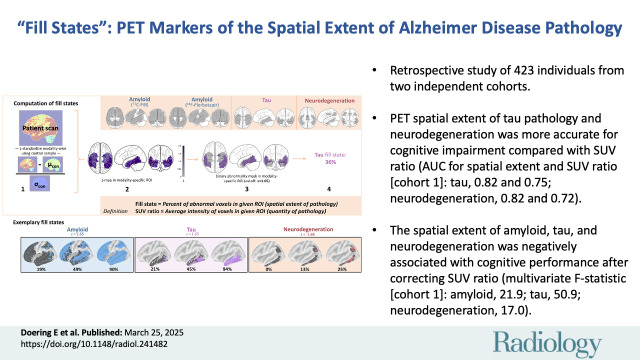

**Summary**

The spatial extent of amyloid, tau, and neurodegeneration, derived from PET imaging, has higher diagnostic performance for cognitive impairment severity compared with standardized uptake value ratios.

**Key Results**

■ In this secondary analysis of prospective studies including 423 individuals from two independent cohorts, the PET-derived spatial extent of tau pathology and neurodegeneration had higher diagnostic performance for cognitive impairment compared with standardized uptake value (SUV) ratio (area under the receiver operating characteristic curve for spatial extent and SUV ratio [cohort 1]: tau, 0.82 and 0.75 [*P* = .002], respectively; neurodegeneration, 0.82 and 0.72 [*P* < .001]).
■ The spatial extent of amyloid, tau, and neurodegeneration was negatively associated with cognitive performance after correcting for SUV ratio (multivariate F-statistic [cohort 1]: amyloid, 21.903; tau, 50.907; neurodegeneration, 17.064; all *P* < .001).

## Introduction

Alzheimer disease (AD) is characterized by amyloid-β plaques, tau tangles, and neurodegeneration, identifiable with PET imaging. The A/T/N (ie, amyloid-tau-neurodegeneration) framework was designed to enhance diagnostic certainty (1) and characterize disease progression (1–3). PET-based quantification of amyloid, tau, or neurodegeneration for disease monitoring typically uses the average standardized uptake value (SUV) ratio of a region of interest (ROI) relative to a reference region (4). Yet, average SUV ratios largely ignore the spatial distribution of pathology, while emerging studies and recommendations emphasize that the spatial extent of pathology may be pivotal for a deeper understanding of symptom variance and disease progression (5–12).

Individuals with widespread pathology may exhibit more severe cognitive impairment compared with individuals with the same quantity of pathology confined to fewer brain regions, potentially allowing compensation by nonaffected brain regions (13). Therefore, spatial extent–based amyloid-tau-neurodegeneration considerations, such as the amount of the brain “filled” with pathology (“fill states”), may provide additional information for monitoring AD progression and screening at-risk individuals, such as patients with subjective cognitive decline (SCD) or mild cognitive impairment (MCI).

Different PET radiotracers enable the quantification of amyloid, tau, and neurodegeneration. The most widely used tracers to quantify amyloid are carbon 11 (^11^C) Pittsburgh compound B and fluorine 18 (^18^F) florbetapir, commonly known as ^18^F-AV45. ^18^F-flortaucipir, also referred to as ^18^F-AV-1451, is currently the only Food and Drug Administration–approved compound to measure tau. Finally, ^18^F-fluorodeoxyglucose uptake is an established measurement for neurodegeneration. Patterns of decreased brain perfusion, as measured using the early uptake phase of various PET tracers (including ^18^F-flortaucipir), have been demonstrated to be highly similar to patterns of hypometabolism derived from ^18^F-fluorodeoxyglucose PET, and, thus, can serve as an indirect measure of neurodegeneration (14,15). To propose spatial extent as a biomarker for AD monitoring, it should consistently correlate with disease progression across clinical sites, potentially using different PET radiotracers.

The aim of this study was to examine the clinical utility and increase the accessibility of PET-derived fill states as markers of spatial extent. To test clinical utility, the efficacy of fill states and the reference standard SUV ratio in reflecting stages of cognitive impairment, from SCD to dementia, as well as cognitive impairment severity (ie, performance on cognitive tests), were compared. To increase accessibility in clinical routine, a convolutional neural network (CNN) was trained to estimate fill states, which circumvents the reliance of the original approach on a control sample. This research seeks to advance PET scan interpretations for AD progression, offering potential benefits for disease staging and monitoring in clinical settings and trials.

## Materials and Methods

This secondary analysis of two prospective studies utilized data from two independent datasets: the Alzheimer’s Disease Neuroimaging Initiative (ADNI) (ClinicalTrials.gov identifier NCT00106899) and the Tau Propagation over Time study (T-POT). Ethical approval for T-POT was granted by the University of Cologne (approval no. 15–194). Written informed consent was obtained from each participant, adhering to the Declaration of Helsinki guidelines. The ^18^F-flortaucipir precursor for T-POT was provided by Avid/Lilly. The authors had full control of the data.

### Datasets

**Figure 1:**
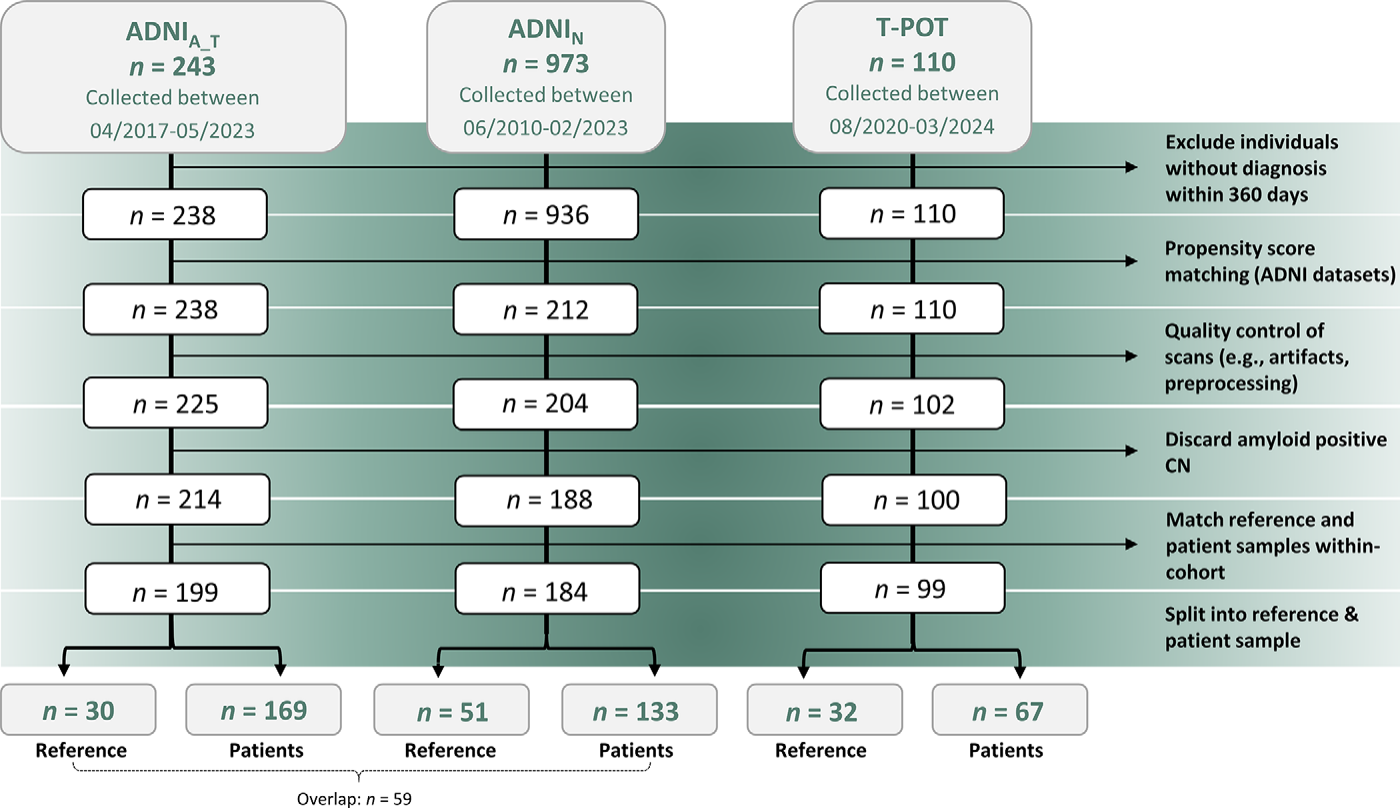
Flow diagram of study sample selection. The Alzheimer’s Disease Neuroimaging Initiative (ADNI) cohort was divided into two groups: the ADNI_A_T_ group had amyloid and tau PET scans available, and the ADNI_N_ group had fluorine 18 fluorodeoxyglucose PET scans available. CN = cognitively normal, T-POT = Tau Propagation over Time study.

Participants included patients with SCD, MCI, and dementia and age- and sex-matched controls (ie, amyloid-negative, cognitively normal individuals) from ADNI (data collected from June 2010 to May 2023) and T-POT (data collected from August 2020 to March 2024). Inclusion in ADNI required an ^18^F-florbetapir (amyloid) and at least one additional (^18^F-flortaucipir [tau] or ^18^F-fluorodeoxyglucose [neurodegeneration]) PET scan, acquired within 180 days. T-POT participants needed an ^11^C Pittsburgh compound B (amyloid) and a dynamic ^18^F-flortaucipir (tau, neurodegeneration) PET scan, acquired within 180 days (Appendix S1, section 1.1). Exclusion criteria for both cohorts were (*a*) no clinical diagnosis within 360 days from the PET examination (*n* = 11); (*b*) artifacts on PET scans (*n* = 29; performed by E.D. [5 years of experience]); and (*c*) amyloid positivity in controls (*n* = 29) (Fig 1).

ADNI included two groups: ADNI_A_T_, individuals who received ^18^F-florbetapir (amyloid) and ^18^F-flortaucipir (tau) (30 controls, 169 patients), and ADNI_N_, individuals who underwent ^18^F-florbetapir and ^18^F-fluorodeoxyglucose PET examinations (neurodegeneration) (51 controls, 133 patients). The datasets were matched using propensity score matching based on age, sex, ^18^F-florbetapir SUV ratios, and diagnostic group (overlap between datasets: 59 patients with triple PET examinations). ^18^F-florbetapir PET was only evaluated from the larger cohort (ADNI_A_T_). T-POT included 99 individuals (32 controls, 67 patients). Individuals without clinically measurable cognitive impairment were considered to have SCD if they had self-reported memory concerns (16), and cognitively normal otherwise. Patients with MCI and dementia (“probable AD” type) received a clinical diagnosis following established criteria without using biomarker information (17).

### Image Acquisition and Analysis

PET scans were spatially and intensity-normalized to yield SUV ratio maps (reference regions for intensity standardization: amyloid and neurodegeneration, whole cerebellum [14,18]; tau, inferior cerebellum [4]; details in Appendix S1, section 1.2). From SUV ratio maps, with use of Python 3.10, two markers of pathology were computed: (*a*) fill states (percentage of abnormal voxels in ROIs; spatial extent) and (*b*) SUV ratio (average voxel intensity in ROIs; quantity).

**Figure 2:**
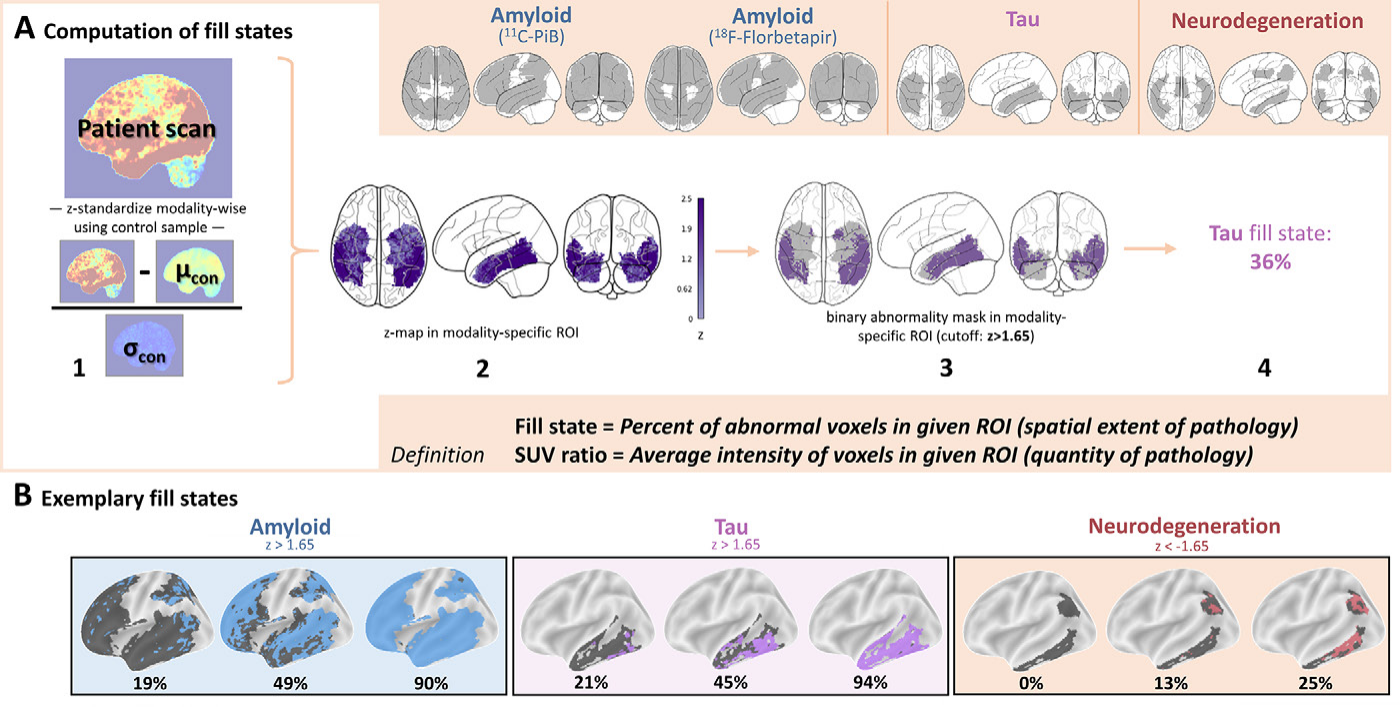
Schematic illustrates how fill states are derived. **(A)** Derivation of an exemplary tau fill state. Note that different meta–regions of interest (ROIs) were recommended for the assessment of amyloid PET markers from carbon 11 Pittsburgh compound B (^11^C-PiB) and fluorine 18 (^18^F) florbetapir (Appendix S1, section 1.3). The bottom beige box provides definitions for the two distinct concepts of fill states and standardized uptake value (SUV) ratios. **(B)** Surface plots corresponding to exemplary fill states for amyloid, tau, and neurodegeneration. The gray-shaded area indicates the meta-ROI, and the colored area indicates abnormal voxels. Color coding: blue = amyloid, purple = tau, red = neurodegeneration. Con = control.

Tracer-specific composite ROIs, where pathologic abnormalities are commonly exhibited (4,19) (Appendix S1, section 1.3), were used to compute these markers. Fill states were calculated as follows (Fig 2A): First, patients’ SUV ratio maps were *z*-standardized using the controls as reference. Second, these *z*-maps were masked using tracer-specific ROIs. Third, each voxel was classified as positive or negative using a *z*-score cutoff of greater than 1.65 (corresponding to *P* < .05 [one-tailed]) for amyloid and tau and less than −1.65 for neurodegeneration (20). Amyloid, tau, and neurodegeneration fill states were computed as the percentage of abnormal voxels in the meta-ROIs. To assess the methodologic robustness of fill states, we performed additional analyses (ie, intensity of abnormal voxels in an ROI and fill states derived from whole-brain gray matter, different reference regions, or different *z*-score cutoffs [Appendix S1, sections 1.4–1.5]).

### Statistical Analysis

Group comparisons and correlations of fill states and SUV ratios with cognitive impairment used nonparametric tests for nonnormal data (Appendix S2, section 2.1). Three levels of significance were considered, namely α = .05, Bonferroni-corrected α (Appendix S1, section 1.6), and α = .001. Analyses were conducted independently in the ADNI and T-POT cohorts.

To compare fill states and SUV ratios between groups (SCD, MCI, and dementia), Kruskal-Wallis *H *tests were computed with post hoc Mann-Whitney *U* tests using Python 3.10. Diagnostic performance in distinguishing cognitive impairment (MCI and dementia) from no impairment (SCD) was assessed using area under the receiver operating characteristic curve (AUC) analysis with DeLong tests in RStudio 4.3.2 (R Foundation).

Partial Spearman correlations in Python 3.10 were used to examine the relationship of fill states and SUV ratios with cognitive performance (Mini Mental State Examination, memory, executive function [Appendix S1, section 1.7]). Correlations were corrected for age, sex, and education. Next, multivariate regression analysis was performed in SPSS Statistics 27 (IBM) to assess the unique effects of fill states, SUV ratios, and covariates on cognitive performance. Specifically, for amyloid (model 1), tau (model 2), and neurodegeneration (model 3), this was modeled as

$${\text{cognitive perfromance = β}}_{\text{0}}{\text{ + β}}_{\text{1}}{\text{Fill state + β}}_{\text{2}}{\text{SUV ratio + β}}_{\text{3}}{\text{Age + β}}_{\text{4}}{\text{Sex + β}}_{\text{5}}\text{Education + ε},$$

wherein all available cognitive performance measures were used as dependent variables and ε represents the residuals. Finally, in model 4, all fill states and the covariates were used as independent variables to identify the unique information of individual fill states (further information: Appendix S1, section 1.8). With ADNI data, model 4 was conducted using the 59 individuals with triple PET examinations and available cognitive scores. Post hoc univariate regression analysis (using each cognitive performance score separately as dependent variable) was conducted to evaluate the effects on each dependent variable individually. To meet the assumptions of multivariate and univariate regression, cognitive performance scores were reflected and square-root-transformed in the ADNI cohorts (Appendix S1, section 1.8). All statistical analyses were repeated in amyloid-positive individuals from the ADNI cohort.

### CNN for Fill State Estimation

The computation of fill states is contingent on the existence of a control sample, which is an evident disadvantage. Therefore, a CNN was trained on 70% of ADNI data to estimate fill states from PET scans directly, in absence of controls (Appendix S1, section 1.9). Performance (mean absolute error, *r*^2^) was evaluated on the remaining ADNI (test set) and all T-POT (external test set) data. Finally, clinical validity was tested by correlating ground truth and CNN-estimated fill states with cognitive performance using partial Spearman correlations (as described in the previous section).

## Results

### Dataset Characteristics

This study included data from a total of 423 individuals from three cohorts (ADNI_A_T_ [*n* = 199; mean age, 71 years ± 6.9 {SD}; 55% female], ADNI_N_ [*n* = 184; mean age, 72 years ± 6.8; 49% female], and T-POT [*n* = 99; mean age, 66 years ± 8.7; 64% female]) (Fig 1). Within each cohort, patients and controls were matched for age and sex (Table 1).

**Table 1:**
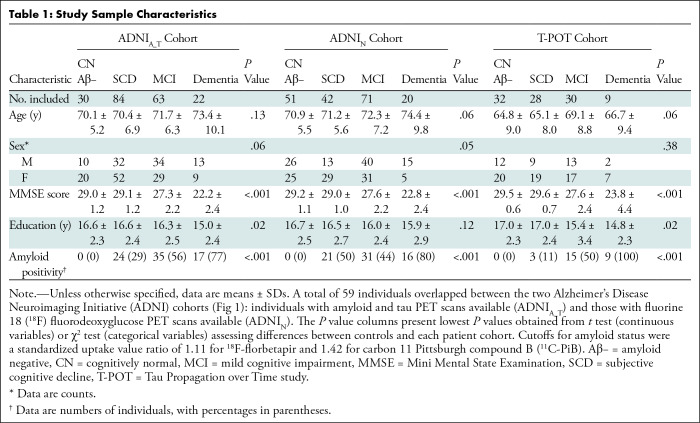
Study Sample Characteristics

### Fill States Differentiate Stages of Cognitive Impairment

**Figure 3:**
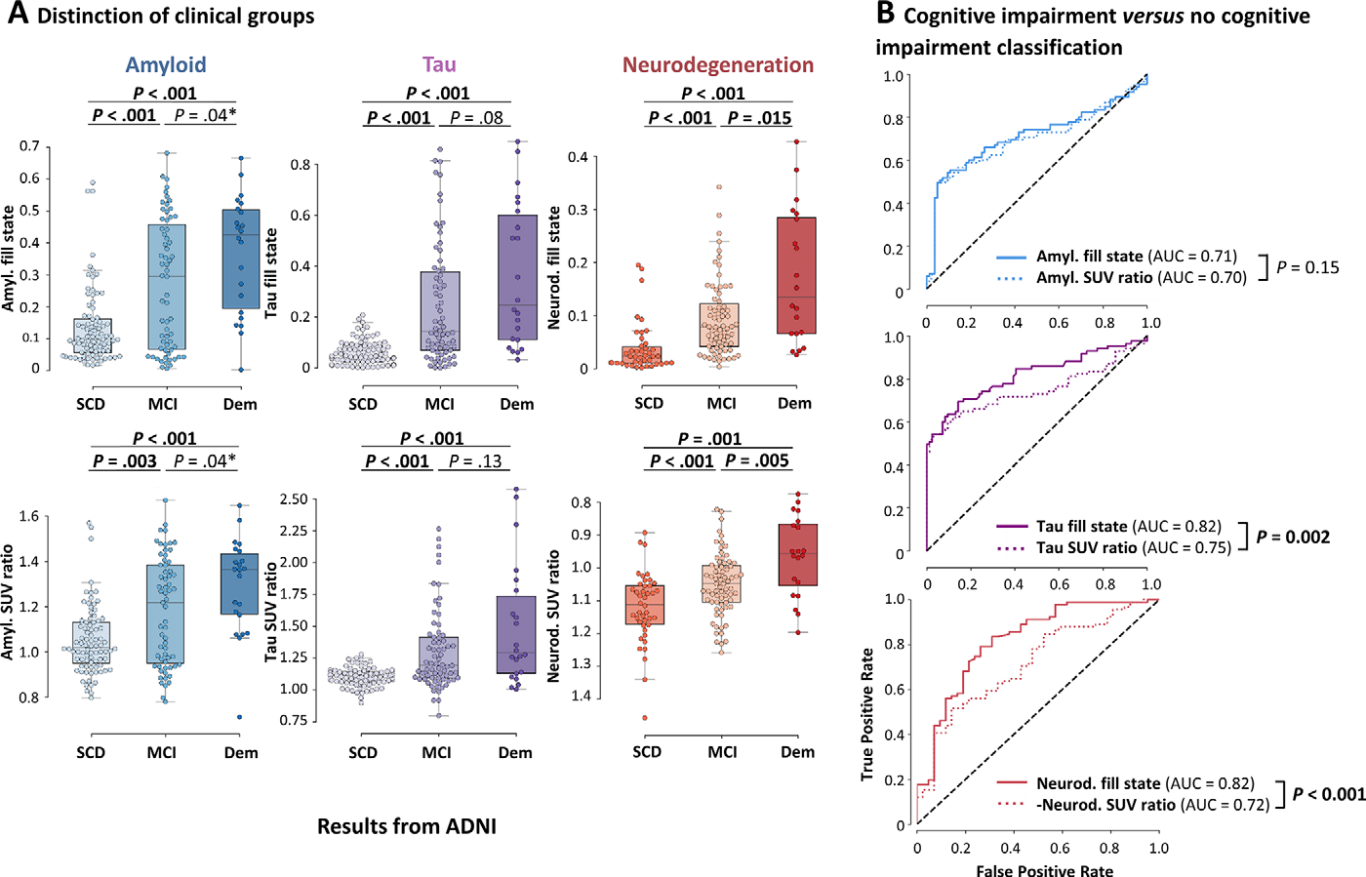
Group comparisons (Alzheimer’s Disease Neuroimaging Initiative [ADNI] cohorts). **(A)** Box plots and group comparisons (Mann-Whitney *U* tests) of fill states (top) and standardized uptake value (SUV) ratios (bottom). The box plots show that higher amyloid (amyl.), tau, and neurodegeneration (neurod.) fill states were directly associated with higher stages of cognitive impairment. The y-axis in the neurodegeneration SUV ratio plot was inverted for interpretability (higher neurodegeneration fill states and lower neurodegeneration SUV ratios would be expected to be associated with higher stages of cognitive impairment). Dots are individual data points (swarmplot), box borders indicate the IQR, the midline represents the median, and whiskers extend to farthest point within the IQR. **(B)** Receiver operating characteristic curve analysis for diagnostic performance in classifying cognitive impairment (mild cognitive impairment [MCI] and dementia [Dem]) versus subjective cognitive decline (SCD) using fill states (solid lines) or SUV ratios (dotted lines). The receiver operating characteristic curves show that tau and neurodegeneration fill states had higher diagnostic performance compared with SUV ratios. Differences in diagnostic performance were assessed using the DeLong test. *P* values in bold are significant after Bonferroni correction (α = .05/3 = .017 for **A** [three groups were compared] and **B** [fill states and SUV ratios were compared in three modalities] [Appendix S1, section 1.6]). * = Significant before Bonferroni correction. Color coding: blue = amyloid, purple = tau, red = neurodegeneration. AUC = area under the receiver operating characteristic curve.

Fill state and SUV ratio distributions were nonnormal, and the two markers of pathology were strongly correlated (Appendix S2, sections 2.1–2.2). In both the ADNI and T-POT cohorts, the Kruskal-Wallis *H *test revealed that all fill states and amyloid and tau SUV ratios differed across groups and cohorts (all *P* < .001) (Table 2; Figs 3A, S3A). Neurodegeneration SUV ratios were significantly different between groups in the ADNI cohort (*P* < .001) but not the T-POT cohort (*P* = .05). Post hoc paired Mann-Whitney *U* tests revealed that all fill states were higher in patients with MCI and dementia compared with those with SCD across cohorts, even after Bonferroni correction.

**Table 2:**
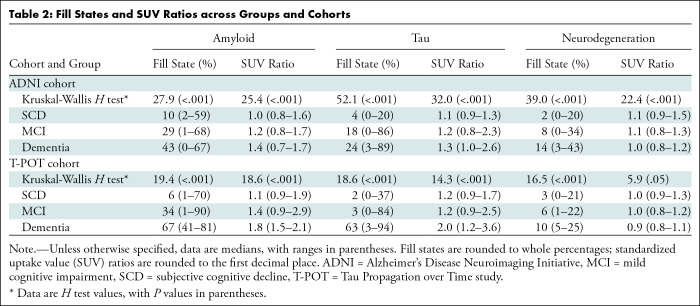
Fill States and SUV Ratios across Groups and Cohorts

Across cohorts, fill states showed high diagnostic performance in distinguishing patients with and without cognitive impairment (AUCs in ADNI for amyloid, tau, and neurodegeneration: 0.71, 0.82, and 0.82, respectively; AUCs in T-POT: 0.79, 0.75, and 0.77). Tau and neurodegeneration fill states had a higher diagnostic performance compared with SUV ratio–based distinction (AUCs in ADNI: 0.70, 0.75, and 0.72; AUCs in T-POT: 0.78, 0.70, and 0.65) according to the DeLong test in both ADNI and T-POT (tau: *P* < .05; neurodegeneration: *P* < .017) (Table 3; Figs 3B, S3B).

**Table 3:**
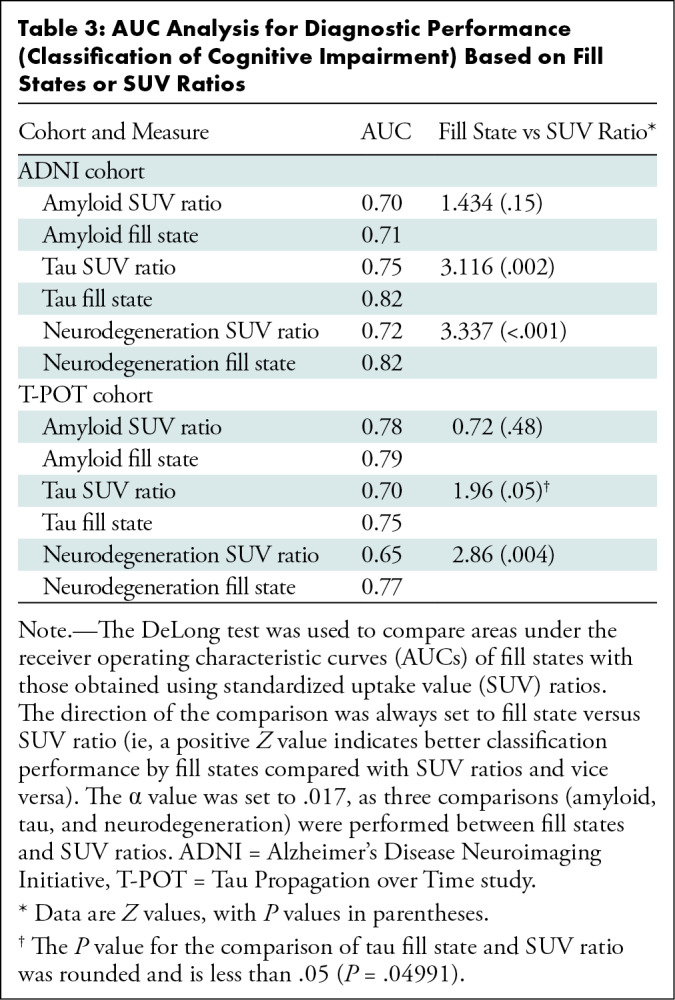
AUC Analysis for Diagnostic Performance (Classification of Cognitive Impairment) Based on Fill States or SUV Ratios

### Fill States Relate to the Severity of Cognitive Impairment

**Figure 4:**
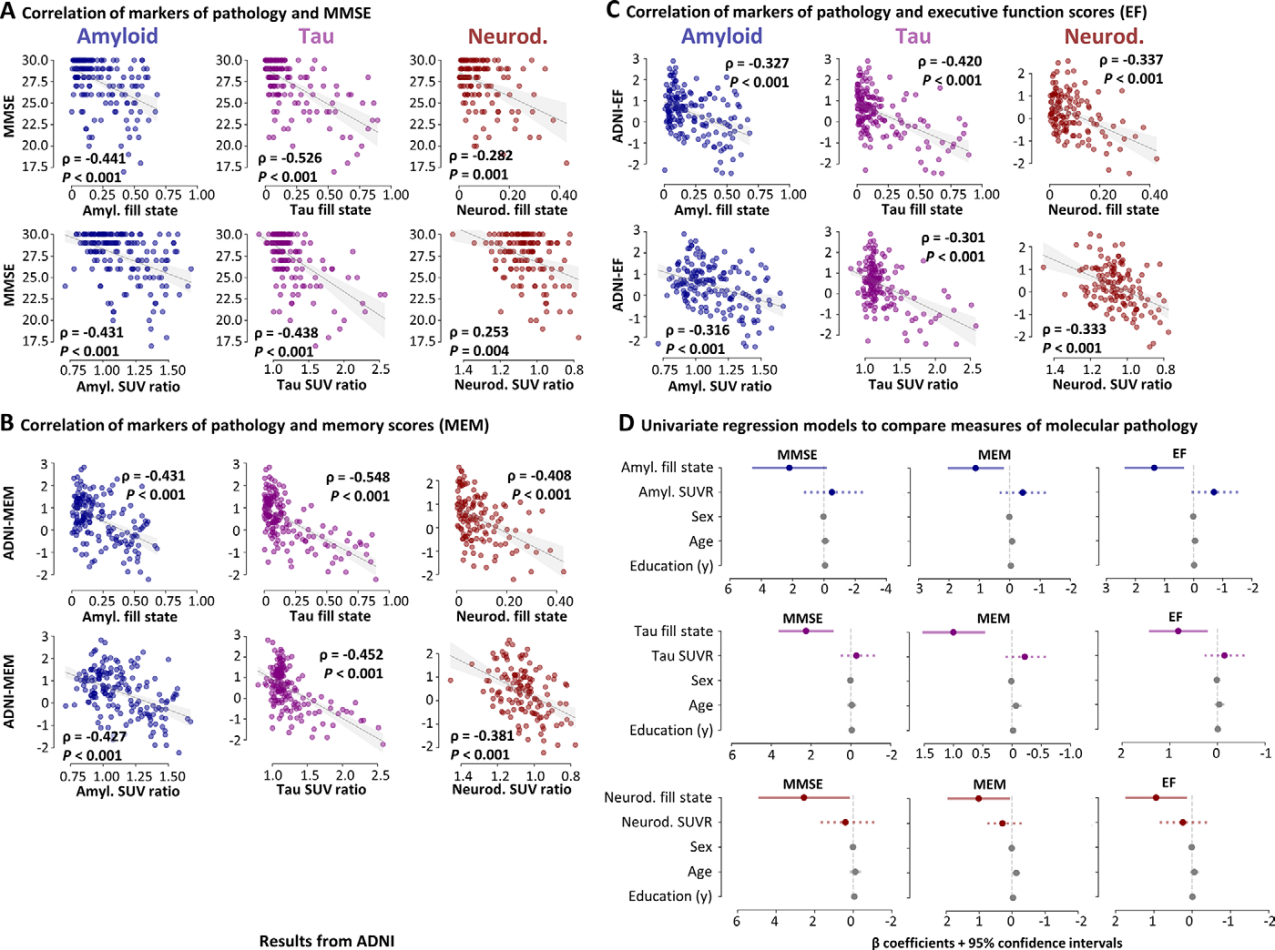
Association of memory performance and markers of pathology (models 1–3, Alzheimer’s Disease Neuroimaging Initiative [ADNI] cohort). **(A–C)** Scatterplots of Mini Mental State Examination (MMSE) scores, memory scores (MEM), and executive function scores (EF) by markers of pathology. The scatterplots show that higher levels of pathology, as indicated by fill states (top) or standardized uptake value (SUV) ratios (bottom), are correlated with more severe cognitive impairment. Correlation strength is indicated as partial Spearman rhos, corrected for age, sex, and education. All correlations were significant after Bonferroni correction (α = .05/3 = .017). For clarity, higher levels of pathology are represented as an increase in the x-axis (ie, the x-axis was inverted for neurodegeneration SUV ratios). The gray line and shaded area represent the regression line and 95% CI, respectively. **(D)** Univariate regression results of cognitive performance by markers of pathology (in color) and covariates (gray). Colored solid lines represent fill states, colored dotted lines represent SUV ratio, and gray solid lines represent the remaining independent variables, respectively. The vertical line represents β = 0. The coefficient plots depict β coefficients for each of the dependent variables separately (left, MMSE scores; middle, memory scores; right, executive function scores) to illustrate the different effects of the predictors on cognition after consideration of the remaining variables. The coefficient plots show that higher fill states, but not SUV ratio measures, were associated with worse cognitive performance scores after accounting for the remaining predictors. Amyloid fill states were not associated with MMSE scores after accounting for the remaining predictors. The x-axis of all coefficient plots was inverted to facilitate interpretability and comparison with Tau Propagation over Time study data (Fig S4). The sign of the coefficients and CIs is inverted in ADNI regression models due to data transformation (ie, a negative association between a marker of pathology and cognitive performance yields a positive β value in regression results) (Appendix S1, section 1.8). The sign of the β coefficient and CI of neurodegeneration SUV ratios was re-inverted for plotting to allow comparability with neurodegeneration fill states: Higher neurodegeneration fill states and lower neurodegeneration SUV ratios would be associated with more severe symptoms. Color coding: blue = amyloid (amyl.); purple = tau; red = neurodegeneration (neurod.). SUVR = SUV ratio.

Partial Spearman correlation analysis showed that fill states and SUV ratios were correlated with all cognitive test scores (fill states: all *P* < .001; SUV ratio: all *P* < .017) (Figs 4A–4C, S4A, S4B), such that more advanced pathology related to more severe cognitive impairment. In multivariate models 1–3, each fill state remained negatively associated with multivariate cognitive performance after adjustment for the remaining variables, including the corresponding SUV ratio across cohorts (Table 4; Appendix S2, section 2.3). In ADNI univariate models, tau and neurodegeneration fill states remained negatively associated with all cognitive scores after SUV ratio and covariate adjustment (tau: all *P* < .017; neurodegeneration: all *P* < .05) (Fig 4D). In T-POT univariate models, both higher tau and neurodegeneration fill states were associated with worse memory performance (tau: *P* = .01; neurodegeneration: *P* < .001), but not Mini Mental State Examination scores (Fig S4C). None of the SUV ratio measures were significant in these models (ie, after adjustment for fill states and covariates). These results indicate that higher tau and neurodegeneration fill states consistently capture variance in memory dysfunction beyond SUV ratios.

**Table 4:**
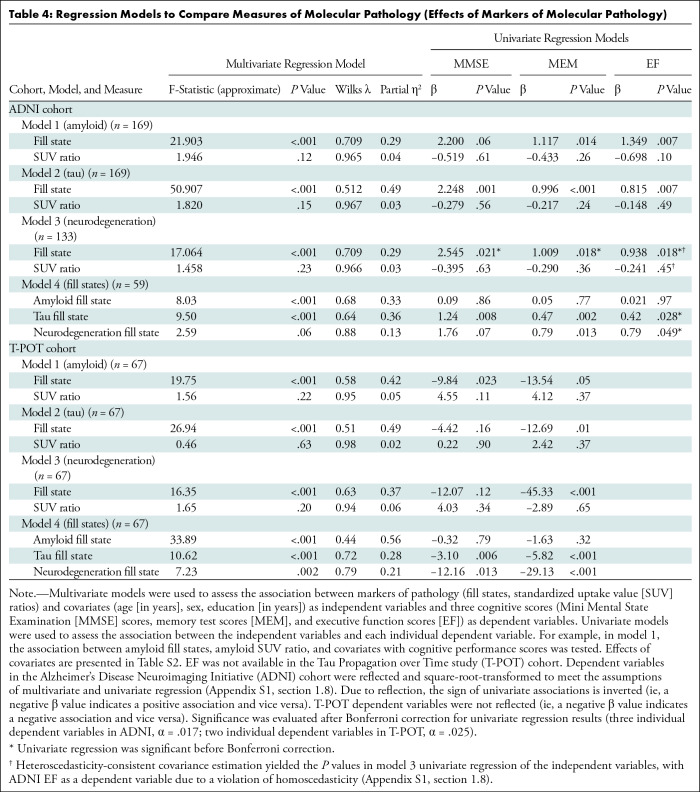
Regression Models to Compare Measures of Molecular Pathology (Effects of Markers of Molecular Pathology)

**Figure 5:**
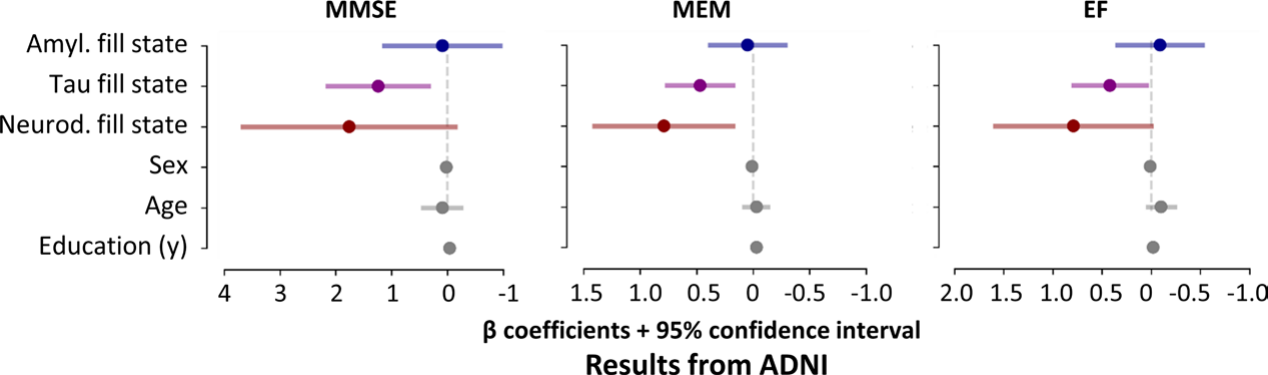
Association of memory performance and fill states (model 4, Alzheimer’s Disease Neuroimaging Initiative [ADNI] cohort). Univariate regression results of cognitive performance by markers of pathology (in color) and covariates (gray). Solid horizontal lines represent the independent variables. Vertical lines represent β = 0. The coefficient plots depict β coefficients from univariate regression models for each of the dependent variables separately (left, Mini Mental State Examination [MMSE] scores; middle, memory scores [MEM]; right, executive function scores [EF]) to illustrate the different effects of the predictors on cognition after consideration of the remaining variables. The coefficient plots show that tau fill states were associated with all cognitive scores after adjustment for the other fill states and covariates. Amyloid (amyl.) fill states were not associated with univariate cognitive performance after adjustment for remaining fill states and covariates. Neurodegeneration (neurod.) fill states were associated with memory scores after adjustment for the remaining fill states and covariates, but not with MMSE or executive function. Color coding: blue = amyloid, purple = tau, red = neurodegeneration.

Finally, all fill states (amyloid, tau, and neurodegeneration) were entered alongside the covariates in model 4. In the multivariate models, amyloid and tau fill states were negatively associated with cognitive performance across cohorts after adjustment for remaining fill states and predictors (all *P* < .001) (Table 4). Neurodegeneration fill states were also negatively associated with cognitive performance in T-POT (*P* = .002), while there was no evidence of this effect in the ADNI cohort (*P* = .06). In model 4 univariate models, tau fill states were negatively associated with all cognitive scores, and neurodegeneration fill states with memory scores across cohorts (all *P* < .05) (Figs 5, S5). Amyloid fill states were not associated with any individual cognitive score; in other words, they only showed a global (multivariate) but not a domain-specific (univariate) association with cognitive performance beyond tau and neurodegeneration. In summary, all fill states provided some complementary information to each other.

### Fill States Are Robust against Methodologic Variation

The diagnostic performance of fill states remained high (AUC ≥0.70) when computed using different reference regions (Fig S6) or different *z*-score cutoffs (Fig S7). Except for neurodegeneration fill states in T-POT, fill states computed in whole-brain gray matter instead of meta-ROIs also displayed high diagnostic performance (Fig S8). Consideration of the average intensity of abnormal voxels did not yield higher accuracies compared with fill states alone (Fig S9).

### Fill States Outperform SUV Ratio in Amyloid-positive Individuals

In amyloid-positive individuals in the ADNI cohort (76 in ADNI_A_T_ and 68 in ADNI_N_), fill states reliably reflected differences in stages (Kruskal-Wallis *H *test; all *P* < .001) (Table S3) and severity of cognitive impairment (partial Spearman correlations; all *P* < .017) (Table S4). All fill states demonstrated high diagnostic performance (all AUCs ≥0.81), with neurodegeneration fill states yielding higher AUC (AUC = 0.84) for identifying cognitive impairment than SUV ratios (AUC = 0.78; *P* = .03 [DeLong test]) (Table S5). Finally, in multivariate regression analysis, all fill states remained negatively associated with cognitive performance after adjustment for SUV ratios and covariates (all *P* < .001), whereas SUV ratios showed no evidence of an association after fill state and covariate adjustment (Table S6).

### Fill States Can Be Estimated from PET Scans with Use of a CNN

**Figure 6:**
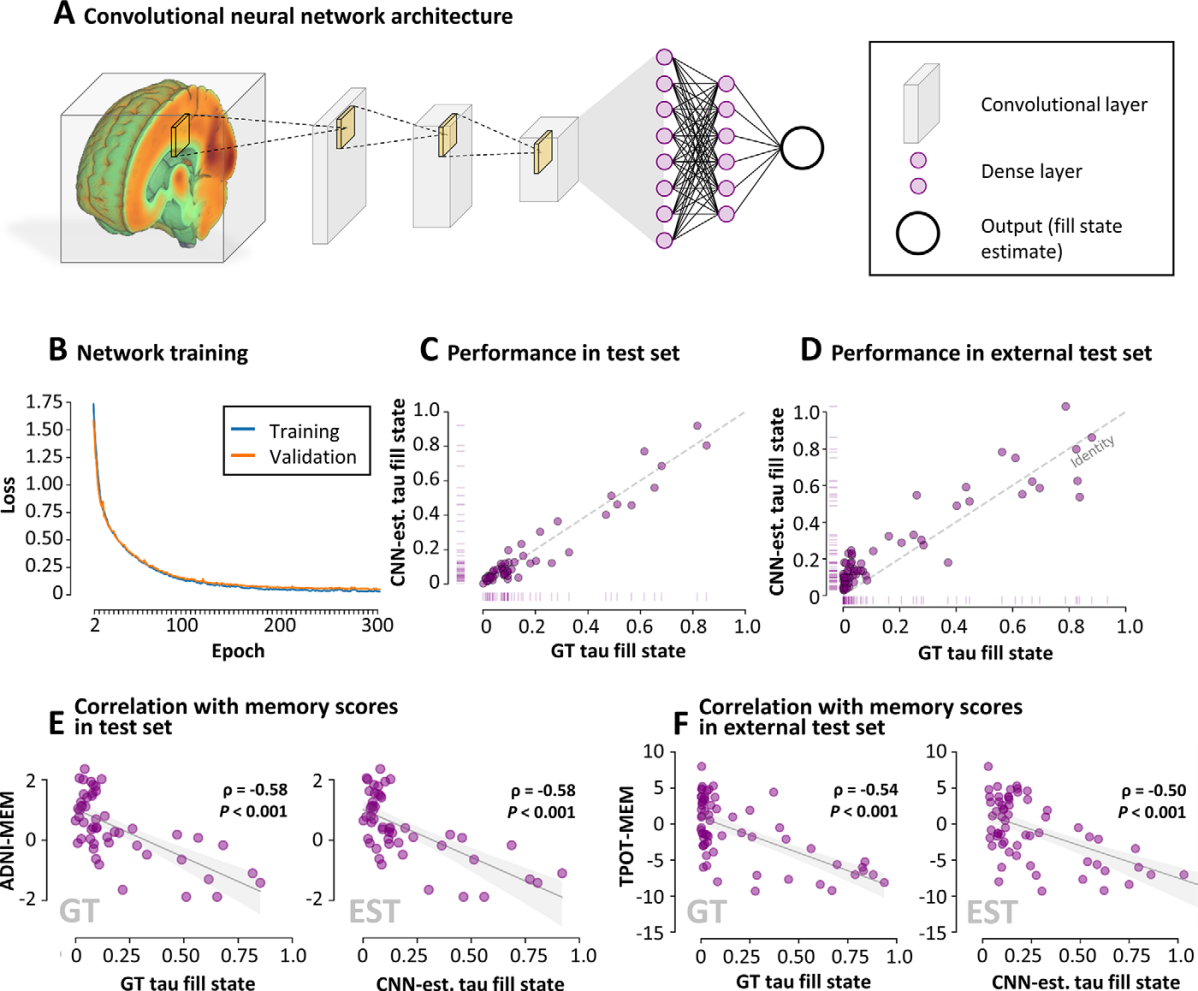
Convolutional neural network (CNN) for the estimation of fill states from PET scans, exemplary for tau fill states. **(A)** Diagram shows architecture of the CNN (Appendix S1, section 1.9). **(B)** Line plot of network training and validation loss across epochs. The network showed consistent learning for tau fill states. Performance in the first epoch was removed for better visualization. **(C)** Scatterplot shows that the performance of tau fill state estimation in the test set (Alzheimer’s Disease Neuroimaging Initiative [ADNI] cohort with amyloid and tau PET scans available [ADNI_A_T_]) was highly accurate. **(D)** Scatterplot shows that the performance of tau fill state estimation in the external test set (Tau Propagation over Time study [T-POT] cohort) was also accurate. Two fill states were predicted to be over 1.0 (= 100%), of which one (value, 1.54) is not depicted for interpretability. Dashed line indicates identity. **(E)** Scatterplot shows that the correlation of ground truth (GT) fill states and memory scores (MEM) was highly similar to the correlation of CNN-estimated (est) fill states and memory scores in the test set (ADNI_A_T_ cohort). Spearman rhos of ground truth fill states differ from previous analysis, as the test set was only a subsample of the entire ADNI_A_T_ cohort. **(F)** Scatterplots show that the correlation of ground truth fill states and memory scores was also highly similar to the correlation of CNN-estimated fill states and memory scores in the external test set (T-POT cohort). The gray line and shaded area depict the regression line and 95% CI, respectively. CIs for all correlations between the ground truth and CNN-estimated fill states are presented in Figure S11.

Amyloid and tau fill states were accurately estimated by a CNN in the test set, while neurodegeneration fill state estimates showed poor explanation of variance (mean absolute error for amyloid, tau, and neurodegeneration: 5.1%, 4.3%, and 4.8%, respectively; *r*^2^: 0.86, 0.93, and 0.46) (Figs 6, S10). Similarly, amyloid and tau but not neurodegeneration fill states were reliably estimated in the external test set (mean absolute error: 10.5%, 10.9%, and 3.5%; *r*^2^: 0.78, 0.74, and 0.34), although the CNN systematically overestimated lower tau fill states (<20%). In the test and external test sets, CNN-estimated amyloid and tau fill states were correlated with all cognitive scores, and neurodegeneration fill states with most cognitive scores (Figs 6E, 6F, S11). This methodologic approach supports the idea that amyloid and tau fill states can be quantified without requiring controls using the proposed CNN.

## Discussion

By means of PET-based quantification of Alzheimer disease (AD) pathology using standardized uptake value (SUV) ratios, a crucial pathophysiologic aspect of AD progression—the spatial extent—is overlooked (7,21,22). Herein, we propose “fill states” as markers representing the spatial extent of amyloid, tau, and neurodegeneration. Fill states showed better performance than reference-standard SUV ratios in characterizing stages and severity of cognitive impairment. Finally, amyloid and tau fill states could be estimated from PET scans with use of a convolutional neural network, improving accessibility of our proposed marker of AD progression.

PET tracer uptake variability is not isotropic throughout the brain but differs between small brain regions or voxels already in the normal population. Average SUV ratios in large ROIs do not control for these differential local variabilities (23,24). Fill states represent simple yet effective continuous biomarkers for the abnormality of PET signal, which account for normal variance though standardization based on amyloid-negative cognitively normal individuals. In line with our results, a number of studies have recently suggested that the spatial extent of tau (8–10,12), amyloid (9,11), and neurodegeneration (9) offers relevant information on disease progression. Iaccarino et al (9) reported that the spatial extent of molecular pathology follows the AD-typical order, wherein first amyloid, then tau, then neurodegeneration increases. However, no comparison with SUV ratios was performed. Gérard et al (8) demonstrated that the spatial extent of tau in Braak regions correlates better with cognitive performance than do SUV ratios. Our investigations complement these studies by outlining that the spatial extent of all three AD hallmark pathologic abnormalities is more closely associated with disease progression of AD compared with SUV ratios. Importantly, many previous studies (8,9,12) included only amyloid-positive patients with cognitive impairment in their analyses. While a positive amyloid status indicates a need for closer monitoring, the amyloid threshold to trigger a neurodegenerative cascade may vary by individual (25–27), and nonamyloidogenic pathways may also cause cognitive decline. Herein, we showed that the advantage of fill states over SUV ratios holds not only in amyloid-positive individuals, but also across entire cohorts. This suggests that fill states might potentially be used to detect abnormal amyloid levels more sensitively and may therefore deliver critical progression information on early stages of the disease. Neurodegeneration fill states, sparsely researched thus far, might also generalize well to non-AD dementias. Promisingly for future research endeavors in this context, we showed that our proposed concept is easily adaptable to different ROIs and robust to some methodologic variation. Finally, this study was the first to propose a CNN for the estimation of fill states that avoids dependence of spatial extent measures on a control sample acquired with the same scanner or according to the same protocol. This innovative approach might facilitate the implementation of fill states in clinical practice.

In AD, the accumulation of protein pathology and increase in neurodegeneration is a gradual process (28). First, small and confined regions of the brain are affected. Resulting spatially constrained disruptions of physiologic processes might be compensable by nonaffected brain regions (13). Once pathology spreads more widely, broader network disruptions occur, which are more challenging to compensate for and cause greater cognitive dysfunction (29). It is thus biologically plausible that measuring the spatial extent of pathology, herein accomplished using fill states, closely reflects symptomatic progression. Since fill states reflected cognitive impairment beyond SUV ratios despite their high collinearity, our results suggest that the spatial extent, rather than the overall quantity of pathology, may be the driving force behind cognitive decline in AD. Importantly, clinical trials currently focus on cognitive decline and SUV ratio reduction to monitor the efficacy of emerging antiamyloid medications (30,31). However, paradoxical results were recently reported, wherein cognitive decline was slowed down without a reduction in tau SUV ratios (31). Potentially, assessing fill states might offer a more nuanced understanding of therapeutic impacts in such trials.

Some limitations of the study must be acknowledged. A number of individuals in our control group showed some evidence of tau pathology but were not excluded to reflect heterogeneity of tau pathology in the healthy population outside the clinical AD spectrum (32). Moreover, our analyses used just two of three approved tracers for amyloid and one for tau, requiring validation with more tracers or a cross-tracer method, such as combining fill states with the Centiloid scale. Finally, fill states were only computed for amyloid, tau, and neurodegeneration. Extending them to potentially coexisting (eg, cerebrovascular [33]) pathologic abnormalities may be critical to better understand individual disease progression.

In conclusion, fill states, representing the spatial extent of pathology, may serve as promising new monitoring biomarkers for Alzheimer disease, which reflect disease progression better than reference-standard standardized uptake value ratios. As such, amyloid, tau, and neurodegeneration fill states may have value for improved staging and monitoring, and they could potentially serve as a readout for clinical trials, pending proof of longitudinal efficacy.
